# Efficacy and safety of *Hominis placenta* pharmacopuncture on mild cognitive impairment: Randomized, double blind, placebo-controlled, multi-center trial

**DOI:** 10.1097/MD.0000000000022956

**Published:** 2020-11-13

**Authors:** Yunna Kim, Jae Hyok Lee, In Chul Jung, Yoon Ji Eom, Seung-Hun Cho

**Affiliations:** a College of Korean Medicine; b Research Group of Neuroscience, East-West Medical Research Institute, WHO Collaborating Center; c Department of Clinical Korean Medicine, Graduate School, Kyung Hee University, Seoul; d Department of Neuropsychiatry, College of Korean Medicine, Semyung University; e Department of Oriental Neuropsychiatry, College of Korean Medicine, Daejeon University; f Department of Neuropsychiatry, Daejeon Korean Medicine Hospital of Daejeon University, Daejeon, Republic of Korea.

**Keywords:** hominis placenta, mild cognitive impairment, pharmacopuncture, protocol, randomized controlled trial

## Abstract

**Introduction:**

Mild cognitive impairment (MCI) is cognitive decline which can be observed in a wide range of cognitive domains. It is considered as a prodromal stage of dementia; therefore, strategies for treatment are necessary, but current evidence is limited. Combining the memory enhancing effect of *Hominis placenta (H placenta)* and acupuncture elucidated separately in previous studies, efficacy of *H placenta* pharmacopuncture for treating MCI is anticipated.

**Methods:**

Thirty participants will be recruited. Male and female adults aged 50 to 80 who voluntarily participate in the trial, are diagnosed with MCI according to diagnostic and statistical manual of mental disorders-5 criteria, and have a Clinical Dementia Rating score 0.5 will be enrolled. Participants who meet the criteria will be randomly allocated to either pharmacopuncture group or control group. Participants will undergo *H placenta* pharmacopuncture or saline pharmacopuncture in GV20, ST36, and CV12 twice weekly for 8 weeks and will be evaluated a month after the last treatment. Primary outcome will be difference in mean change of Korean version of Montreal Cognitive Assessment scores between intervention group and control group. Cognition, mood, sleep quality and quality of life will be also assessed using other neuropsychological tests and questionnaires regarding depression, anxiety, sleep and quality of life.

**Discussion:**

Evaluating the efficacy and safety data obtained by assessing diverse aspects of patients with MCI will broaden the scope of MCI management and prevention of dementia progression.

**Trial registration:**

Clinical Research Information Service (KCT0005368), Registered 02 Sep 2020, https://cris.nih.go.kr/cris/en/search/search_result_st01.jsp?seq=16425

## Abstract 2

Supplemental Digital Content is available in the text

## 1 Introduction

Mild cognitive impairment (MCI) is a transitional state from normal aging to dementia. The Diagnostic and Statistical Manual of Mental Disorders (DSM-5) criteria for diagnosis of MCI is that cognitive decline is present in 1 or more cognitive domains (complex attention, executive function, learning and memory, language, perceptual-motor, and social cognition) but does not interfere with independence in daily activities.^[1]^ MCI is considered a prodromal stage of dementia—10% to 15% of patients with MCI develop Alzheimer's disease every year, given a 1% to 2% annual progression rate in the normal population.^[2]^ However, while diverse pharmacological and cognitive interventions are under investigation, the evidence regarding effective therapies for MCI is still limited;^[3,4]^ consequently, as per the established guidelines, pharmacological treatment is only recommended for symptomatic treatment.^[5]^

Pharmacopuncture is a treatment that involves the insertion of medication to be embedded into a specific acupoint. It is a combined form of acupuncture and herbal medicine and is applied for the treatment of diverse diseases including neuropsychiatric diseases. Two randomized control trials showed that pharmacopuncture combined with oral administration of other drugs had better effects on the cognition of patients with Alzheimer disease than oral administration itself or intramuscular injection combined with oral administration.^[6,7]^

*Hominis placenta* (*H placenta*) is human placenta and contains immune substances such as interferons, blood coagulation factors, hormones and their precursors, and cell proliferation factors. In traditional medicine, *H placenta* was used for treating memory loss, agitation, and disorientation, which are now known to be consistent with major symptoms of dementia. Animal experiments revealed that *H placenta* itself or medication containing *H placenta* increased both learning and memory ability and brain-derived neurotrophic factor expression in the brain.^[8–10]^ Recently, several randomized controlled trials and case studies were conducted on the treatment of dementia using oral administration of medication containing *H placenta*.^[11–17]^

The efficacy of *H placenta* when injected into acupoints has been investigated as well. Animal experiments were conducted to evaluate memory-related changes in animal models of Alzheimer's disease using *H placenta* pharmacopuncture. In both an amyloid β (Aβ) injection model and a transgenic animal model, cognition-related behavior was ameliorated, and significant changes were observed in brain tissue.^[18,19]^ 0.1 ml of *H placenta* was injected in CV12 of mice that received intrahippocampal injections of Aβ; subsequently, the mice showed significant changes in stop-through latency and distance movement-through latency in the Morris water maze. In addition, *H placenta* decreased the levels of proinflammatory cytokines, such as IL-1β and TNF-α, oxidative stress markers, and factors related to Aβ and tau pathologies in brain tissue.^[19]^ Injecting *H placenta* in the GV20, CV12, ST36, and HT7 of an animal model of Alzheimer's disease significantly increased time spent around the platform in the Morris water maze test and increased spontaneous alternation in the Y-maze, indicating improvement of memory. It was confirmed that brain-derived neurotrophic factor significantly increased and Aβ decreased compared to corresponding levels in the control group (unpublished data). Intraperitoneally and orally administered *H placenta* in 5XFAD mice improved memory as confirmed using the novel object recognition test, and decreased dendrite density around Aβ plaques was reversed.^[18]^ Human study regarding *H placenta* pharmacopuncture or injection on cognitive disorder was conducted only in a case study. Our previous study showed that an 84-year-old male patient with MCI was treated using *H placenta* pharmacopuncture in GV20, CV12, ST36 once weekly for 1 month and that his score in neuropsychological tests, such as Korean version of the Montreal Cognitive Assessment (MoCA-K), Mini-Mental Status Examination for Dementia Screening (MMSE-DS), and Korean Dementia Rating Scale (K-DRS), improved.^[20]^

Considering results from a series of preclinical studies and a case report, it was considered important to conduct a randomized controlled trial examining whether *H placenta* pharmacopuncture improves memory. Thus, the aim of this study is to investigate the effect of *H placenta* pharmacopuncture versus saline pharmacopuncture on patients with MCI with regard to aspects of memory and accompanying symptoms related to mood, sleep, and quality of life after 8-week administration.

## 2 Methods

### 2.1 Objectives

The aim of this study is to explore the efficacy and safety of *H placenta* pharmacopuncture in patients with MCI. To this end, the trial is designed to evaluate its efficacy on cognitive functions assessed using the MoCA-K, MMSE-DS, K-DRS, Clinical Dementia Rating (CDR), and Global Deterioration Scale (GDS) and to assess mood change, sleep disturbance and quality of life using the Korean version of Beck Depression Inventory-II (K-BDI-II), State-Trait Anxiety Inventory (STAI), State-Trait Anger Expression Inventory (STAXI), Insomnia Severity Index (ISI), Euro Quality of Life - 5 Dimensions (EQ-5D), Euro Quality of Life - visual analog scale (EQ-VAS), and Geriatric Quality of Life scale-Dementia (GQOL-D).

### 2.2 Design

**Figure 1:**
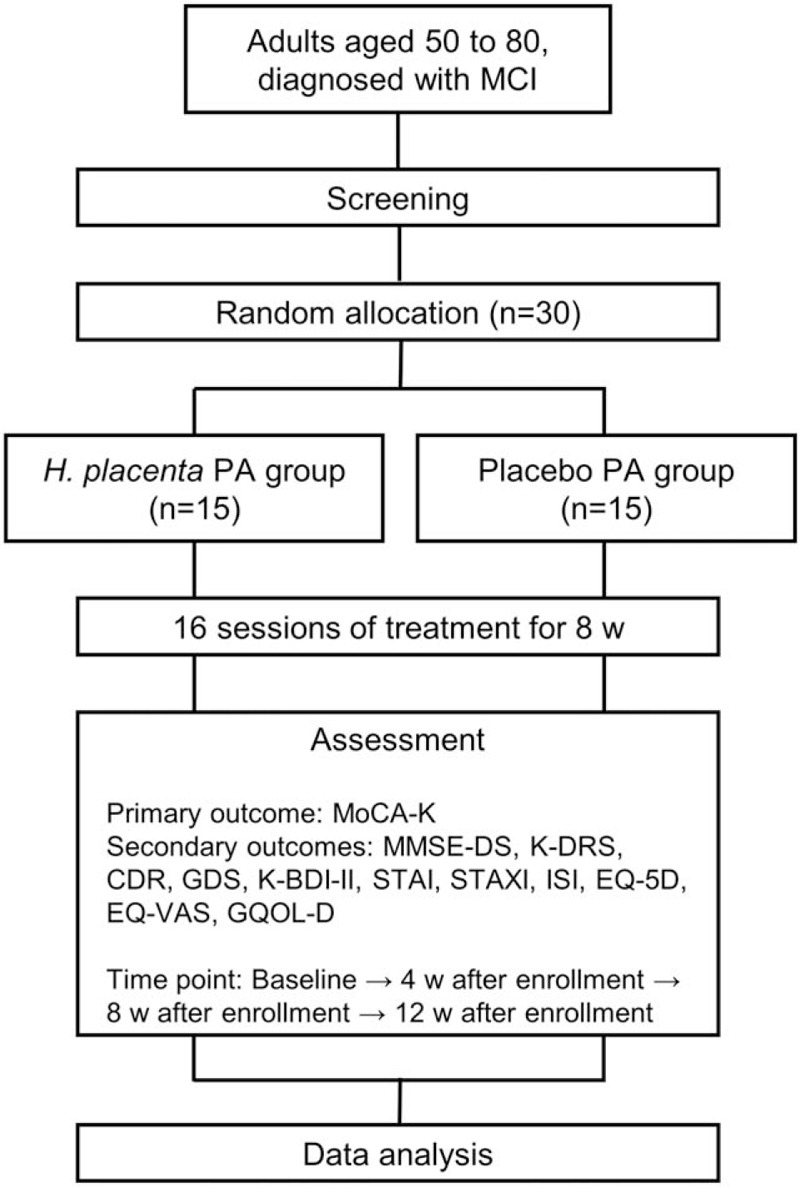
Diagram of the study flow.

This study is a randomized, double-blind, placebo-controlled, 2-center clinical trial to objectively evaluate the efficacy and safety of *H placenta* pharmacopuncture for MCI (Fig. 1). *H placenta* pharmacopuncture or placebo will be administered to participants for 8 weeks and changes in symptoms observed before and after 8 weeks of administration. This trial will be performed in Kyung Hee University Korean Medicine Hospital and Semyung University Korean Medicine Hospital. This protocol is presented in accordance with the Standard Protocol Items: Recommendations for Interventional Trials 2013 statement (see Table, Supplemental file 1, Fig. 2). The organizational structure and responsibilities of the researchers are shown in Supplemental file 2.

**Figure 2:**
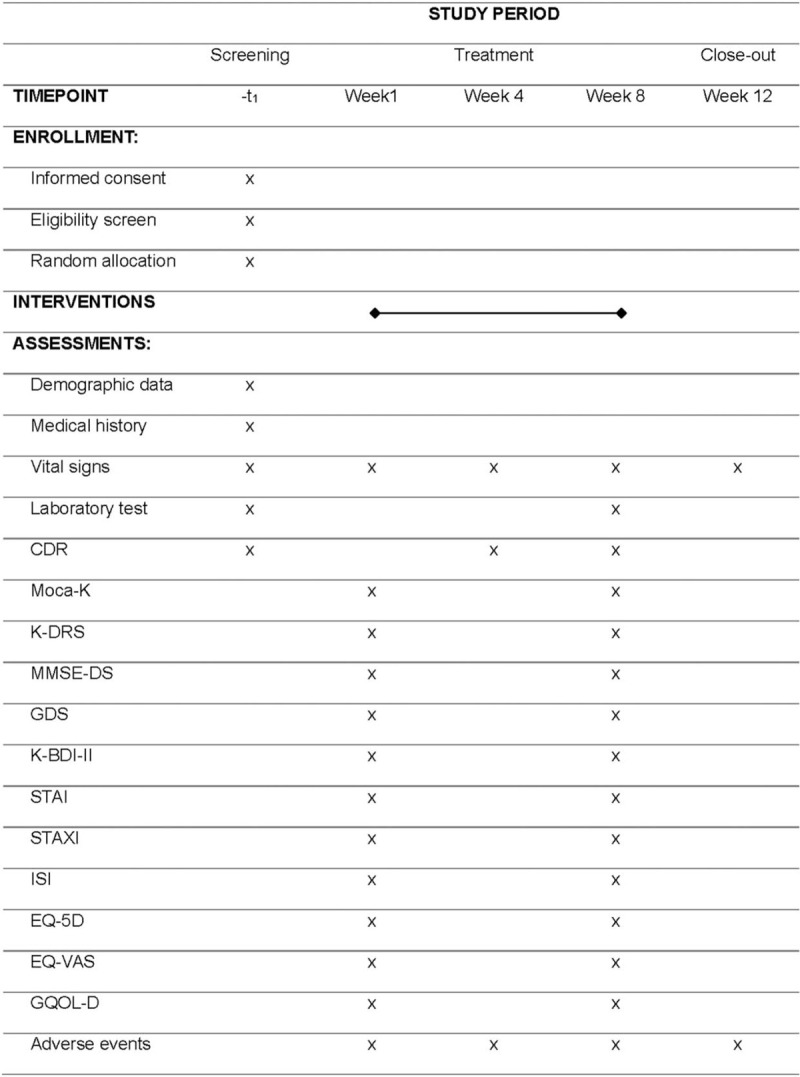
Standard protocol items: recommendations for interventional trials statement. Overview of study process and outcome assessment.

### 2.3 Ethical considerations

The Institutional Review Boards of Kyung Hee University Korean Medicine Hospital and Semyung University Korean Medicine Hospital approved the study (KOMCIRB 2019-10-004 and SMCJH 1912-08, respectively). This trial complies with the Declaration of Helsinki. Korean neuropsychiatrists will explain participant title, purpose, period, method, expected effect, risk, protection of the participants, consent for voluntary participation, and withdrawal of consent using the informed consent form. The participant will voluntarily decide whether to participate or not and will either sign or not sign, respectively, an informed consent form. Protocol amendments will be approved by IRB and these modifications will be updated in protocol registration site or a journal. If the modification results in alteration in their benefit or harm, participants will be notified. The results of the trial will be disseminated via publication in peer-review journals.

### 2.4 Participants

#### 2.4.1 Assessment of eligibility

Participants who voluntarily agree to the trial will be given a subject identification code in order, and undergo a demographic survey, vital sign and laboratory tests, and neuropsychological examinations to determine whether they meet the inclusion/exclusion criteria.

#### 2.4.2 Inclusion criteria

(1) Male or female adult aged 50 to 80.
(2) A person diagnosed with mild neurocognitive impairment based on DSM-5.
(3) A person who can undergo neuropsychological tests and complete questionnaires.
(4) A person who agrees to participate in the trial after receiving explanations regarding the purpose and procedures involved in the clinical trial and has signed the informed consent form by oneself or one's legal representative.
(5) A person with a score of 0.5 on the CDR.

#### 2.4.3 Exclusion criteria

(1) A person with a cranial lesion or brain injury that can cause cognitive decline.
(2) A person with a history of cerebral hemorrhage or cerebral infarction.
(3) A person with brain diseases such as Parkinson disease, epilepsy, and brain cancer.
(4) A person with a present illness or past history of a major psychiatric disorder such as schizophrenia, delusional disorder, depressive disorder, bipolar disorder, or alcohol or substance abuse disorder as diagnosed using DSM-5.
(5) A person who has participated in other clinical trials within the last 1 month.
(6) A person who had acupuncture treatment for cognitive impairment within the last 4 weeks.
(7) A person who is currently taking medication related to dementia (If the person is diagnosed with MCI and is taking dementia-related drugs, he/she may participate in the clinical trial after the wash-out period of 15 days).
(8) A person with a severely destabilizing general medical condition (a doctor in charge will judge the enrollment based on the results of laboratory tests, vital signs, etc).
(9) A person with a clinically serious liver disease or with serum aspartate transaminase and alanine transaminase levels exceeding twice the upper limit of reference value.
(10) A person with chronic renal failure or with serum blood urea nitrogen and creatinine levels exceeding 1.5 times the upper limit of reference value.
(11) Pregnant woman, lactating woman, or woman of childbearing potential who do not use appropriate methods of contraception.
(12) A person who researchers judge to be inappropriate for participation in this clinical trial.
(13) A person who is hypersensitive to *H placenta* and other drugs.
(14) A person with moderate complications other than those involving the heart, liver, and kidney.
(15) A person with psychogenic diseases.

#### 2.4.4 Discontinuation criteria

Participants can be excluded from the study when any of the following conditions are met:

(1) In case the participant withdraws their consent to participate in the trial.
(2) In case the participant violates the inclusion criteria or meets the exclusion criteria.
(3) In case the participant has serious adverse events (AEs), or it is difficult to continue the clinical trial due to AEs.
(4) In case it is judged impossible to continue treatment or observe the participant due to an unexpected disease or accident.
(5) In case the participant requests discontinuation or the investigator determines discontinuation due to a worsening cognitive deficit during the clinical trial.
(6) In case cognitive function deteriorates during the clinical trial and CDR becomes 1 or higher.
(7) In case it is determined that there is an unavoidable reason for the participant to discontinue the trial.
(8) In case overall compliance is less than 70%.

To promote compliance of participants, we plan to send a text message as a reminder a day before the appointment.

### 2.5 Recruitment

Participants will be recruited by posting documents at appropriate places such as bulletin boards in hospitals, community centers, and subways. During the recruitment process, participant anonymity will be maintained to protect personal privacy and ensure confidentiality. In addition, assigned personnel will respond to recruitment inquiries and keep the recruitment data in a space with limited access.

### 2.6 Randomization, allocation concealment, and blinding

30 eligible participants each will be randomly assigned to the pharmacopuncture group and the placebo group in a 1:1 ratio. Block randomization will be performed on participants who meet the inclusion/exclusion criteria. The randomization number will be generated by an independent statistics expert. The generated number will be placed in a lightproof, sealed envelope and kept in a locked chamber that is accessible only to people authorized to be unblinded. After completion, database lockout will be performed, and then the random number table with intervention group or control group labelled either A or B will be provided to the investigator responsible for statistical analysis. After the statistical analysis is completed, the group information marked either A or B will be disclosed.

This clinical trial is designed to be double-blinded by separating the pharmacopuncture practitioner and assessor from the process of transferring *H placenta* from ampule to syringe and controlling the bias as much as possible. To avoid participant bias as much as possible, the syringe used will have a translucent tape on the surface to avoid the participant from noticing the color of the liquid. Participants will be informed that they have been allocated to 1 of 2 groups and receive the treatment in a clinic setting similar to where 1 generally receives treatment in Korean medicine hospitals or clinics. The practitioner will inject the same amount of *H placenta* or saline with the needle at the same acupoints so that both practitioner and participant do not infer allocation. The validity of the blinding will be assessed by an investigator who is not involved in either allocation or performance of the pharmacopuncture treatment. If an emergency occurs during the clinical trial, such as a serious AE (SAE), and it is absolutely necessary to protect the rights and safety of the participant, unblinding can be performed at the request of an investigator. The investigator should then report the details of the need for unblinding to the IRB.

### 2.7 Interventions

Pharmacopuncture treatment will be performed by Korean neuropsychiatrists following the details of the Standards for Reporting Interventions in Clinical Trials for Acupuncture 2010 checklist (Table 1). Each participant will be scheduled to visit the institution twice a week for 8 weeks after screening and receive a total of 16 treatments of *H placenta* pharmacopuncture or placebo pharmacopuncture. 1 mL of *H placenta* or placebo will be injected twice a week into acupoints (GV20, both ST36, CV12). *H placenta* (Unimed Pharmaceutical, Seoul) is a pale-yellow transparent liquid contained in a brown ampoule. The content is 1 mL of *H placenta* extract per 1 mL. It should be stored in the shade in sealed containers at room temperature (1–30°C). Saline will be used as placebo; it is a colorless, clear liquid with a salty flavor. The content is 9 mg sodium chloride in 1 mL. Other drugs and therapies to ameliorate cognitive function are prohibited in principle during the clinical trial period, and in the event of concurrent treatment, the clinical trial director will determine whether the participant will be excluded from the study and the statistical analyses.

**Table 1:** Details of acupuncture intervention (STRICTA).

Item	Detail	Description
Acupuncture rationale	(1a) Style of acupuncture	Pharmacopuncture
	(1b) Reasoning for treatment	The treatment was selected based on previous studies such as animal experiments and a case report.
	(1c) Extent to which treatment varies	None
Details of needling	(2a) Number of needle insertions per subject per session	4
	(2b) Names of points used (unilateral/bilateral)	GV20, CV12, ST36 (bilateral)
	(2c) Depth of insertion	5–10 mm
	(2d) Response sought	None
	(2e) Needle stimulation	None
	(2f) Needle retention time	None
	(2g) Needle type	0.30 mm × 8 mm, single use syringes (Becton, Dickinson and Company, USA)
Treatment regimen	(3a) Number of treatment sessions	16 sessions
	(3b) Frequency and duration of treatment sessions	2 sessions/wk for 8 wk
Other components of treatment	(4a) Details of other interventions administered to the acupuncture group	None
	(4b) Setting and context of treatment	Hospital outpatient department
Practitioner background	(5) Description of participating acupuncturists	Korean neuropsychiatrist or Korean medical doctor with an experience of more than 1-yr under the guidance of a Korean neuropsychiatrist
Control or comparator interventions	(6a) Rationale for the control or comparator in the context of the research question	Saline will be used as placebo control.
	(6b) Precise description of the control or comparator	Saline will be used as placebo control and will be inserted using the same syringe type into the same acupoints.

### 2.8 Outcome measurement

#### 2.8.1 Primary outcome

The primary outcome is the difference in mean change of MoCA-K scores between intervention group and control group. The MoCA-K is a tool that was originally developed by Nasreddin et al.^[21]^ to screen for MCI, which was translated into Korean and validated. It consists of 7 areas to evaluate memory, visuospatial/ executive function, naming, attention, language, abstraction, and orientation. The maximum score is 30 points, and 1 point is added when the participant has been educated for less than 6 years to correct differences in cognition due to academic background. It suggests a cutoff point of 22 or less for screening MCI,^[22]^ which means a decrease in cognitive function. Reliability of the original tool was Cronbach's α =.83^[21]^ and that of the translated tool was Cronbach's α =.81–.84.^[22]^

#### 2.8.2 Secondary outcomes

Secondary outcomes are

(1) differences in mean change of MMSE-DS, K-DRS, CDR, GDS, K-BDI-II, STAI, STAXI, ISI, EQ-5D, EQ-VAS, and GQOL-D scores between intervention group and control group, and
(2) direction of intra-group change of mean of K-DRS, MMSE-DS, MoCA-K, CDR, GDS, K-BDI-II, STAI, STAXI, ISI, EQ-5D, EQ-VAS, and GQOL-D scores in pre- and post-treatment evaluation. K-DRS, MMSE-DS, MoCA-K, GDS, K-BDI-II, STAI, STAXI, ISI, EQ-5D, EQ-VAS, and GQOL-D scores will be assessed at baseline and 8 weeks, and CDR score will be assessed at baseline, 4 weeks, and 8 weeks.

##### 2.8.2.1 K-DRS

The K-DRS^[23]^ is the Korean version of the Dementia Rating Scale,^[24]^ which consists of 5 subtests suitable for examining general cognitive functions: attention, execution, organization, memory, and conceptualization. The score ranges from 0 to 144 points. The correlation with MMSE-K was 0.82, and 2-week test-retest reliability was 0.96.^[23]^ There was a significant performance difference between normal and dementia patients,^[23]^ and neurological validity was supported by structural brain magnetic resonance imaging (MRI) studies.^[25]^

##### 2.8.2.2 MMSE-DS

The MMSE-DS was developed by Han et al (2010) to measure cognitive function in the elderly. It consists of 19 questions about time and place orientation, immediate and delayed recall, concentration, naming, repetition, visuospatial construction, reading and writing, abstract thinking, and judgement. The scores obtained are calculated by adding all the scores obtained based on the 19 questions. The maximum score is 30 points, and the cutoff score varies according to age, gender, and education. The Pearson correlation coefficient between 4-week interval test-retest results was 0.935 (*P* < .001).

##### 2.8.2.3 GDS

GDS is designed to classify the clinical severity of patients who are suspected to have dementia or are diagnosed with dementia. It is a revised version of the GDS designed by Reisberg et al^[27]^ It is advantageous because the practitioner is able to easily determine and judge the severity of dementia in a relatively short time by comparing the degree of cognitive impairment described in each grade with specific examples. Reisberg et al showed that the inter-tester reliability is 0.95.^[27]^

##### 2.8.2.4 CDR

The CDR scale was developed by Hughes et al^[28]^ to measure the degree of cognitive and social function of dementia patients. The Korean version translated by Choi et al^[29]^ will be used in this study. This tool evaluates dementia severity by compiling reports from the patient and the caregiver regarding 6 areas of memory, orientation, judgment and problem solving, community affairs, home and hobbies, and personal care. Each item is rated either 0, 0.5, 1, 2, 3, 4, or 5 points; a higher score means higher severity. There are 2 ways to calculate the scores—by adding all 6 area scores (sum of boxes) or by determining the total CDR score based on the memory item (global score).

##### 2.8.2.5 K-BDI-II

The 21-item BDI was developed by Beck et al^[30]^ to measure the severity of depression. Items of the K-BDI-II are scored on a 4-point Likert scale ranging from 0 to 3 points, with a range of 0 to 63 points in total. A higher score means more severe depression. Depression severity is classified into 4 levels: normal (0–13), mild depression (14–19), moderate depression (20–28), and severe depression (29–63).

##### 2.8.2.6 STAI

It is a measure that can evaluate state anxiety and trait anxiety simultaneously and was developed by Spielberger et al.^[31]^ In order to measure anxiety experience, it evaluates of state anxiety (20 questions) and trait anxiety (20 questions), each on a 4-point scale. The score ranges from 20 to 80, and a higher score means higher anxiety.

##### 2.8.2.7 STAXI

The STAXI is a scale developed by Spielberger^[32]^ and consists of 44 questions that evaluate state anger, trait anger, and anger expression. Each item is measured on a 4-point scale and 5 subscales: state anger, trait anger, anger suppression, anger expression, and anger control.

##### 2.8.2.8 ISI

This is a subjective measure of insomnia developed by Morin (1993), and a self-reporting measure that assesses the type and severity of insomnia, sleep satisfaction, disturbance of daytime function, and sufferings from sleep disorders. There are 7 items, and each item is scored from 0 to 5 points with the total ranging from 0 to 28. Higher scores indicate more severe symptoms of insomnia. The severity of insomnia is classified into 4 levels: normal (0–7), subthreshold insomnia (8–14), moderate insomnia (15–21), and severe insomnia (22–28).

##### 2.8.2.9 EQ-5D, EQ-VAS

Euro Quality of Life (EuroQol) Group developed it as a tool for measuring health-related quality of life.^[34]^ It consists of a description part and evaluation part. The description part has 5 questions that measure the current health status 5 dimensions: mobility, self-care, usual activities, pain/discomfort, and anxiety/depression. Each dimension is rated by 3-level scale, which are no problem (level 1), moderate problem (level 2), and extreme problem (level 3). By combining the items answered from the 5 questions, there are potentially 243 different health states. Evaluation part consists of EQ-5D index and EQ-VAS. EQ-VAS using a visual analogue scale is designed to indicate one's current health status on a vertical line graded from 0 (the worst imaginable health) to 100 (the best imaginable health).

##### 2.8.2.10 GQOL-D

GQOL-D is a scale to measure the quality-of-life of dementia patients, especially Alzheimer disease. It is designed to comprehensively cover all different sub-areas from other quality-of-life tools for the elderly^[35]^ A clinician conducts the assessment by an interview. This scale consists of a total of 15 items, including 13 items that measure physical health, psychological health, social relations, and environment and 2 items that respectively measure overall health and overall life satisfaction. The clinician asks a question and the subject indicates their quality-of-life and satisfaction, on a scale from 1 to 4 (1 = not satisfied, 2 = normal, 3 = satisfied, 4 = very satisfied). The total score is calculated from the sum of responses to each question, and the total score ranges from 15 to 60 points. The total score is converted into a standard score (T score) according to gender and age. The higher T score means the higher subjective quality-of-life and satisfaction of the subject. Conversely, the T score below 35T indicates significantly lower quality-of-life compared to the group of same gender and age.

### 2.9 Sample size calculation

Since there were no previous clinical trials on *H placenta* for this indication, the results of previous studies on herbal medicines using MoCA scores^[36]^ were cited. According to the previous study comparing MoCA scores after herbal medicine or placebo treatment of patients with MCI, calculation using a standard formula^[37]^ yielded a sample size of 10 for each group, with 80% statistical power at 5% level significance (α=0.05, 1-β=0.8). Assuming a dropout rate of 30% considering the 8-week-long administration and follow-up period, the final sample size will be 15 for each group, 30 in total.

### 2.10 Statistical analysis

Among the demographic data, continuous data will be presented as mean and standard deviation, and categorical data will be presented as frequency and percentage. Group comparison of continuous demographic data will use the *t*-test if normality is satisfied in and the Wilcoxon rank sum test if normality is not satisfied. Categorical data will be compared using the Chi-square test or Fisher exact test.

The independent sample *t*-test will be applied for comparison of the difference in the mean change in parameter before and after the clinical trial between intervention group and the control group, and the Wilcoxon rank sum test can be applied when nonparametric analysis is required. In order to compare the difference in the change in trend by each group visit, data will be analyzed using repeated measures analysis of variance. All the statistical tests will adopt 2-tailed testing considering an alpha value of 0.05 with 80% power. Intent to treat will be mainly performed, but per-protocol will also be performed as a supplement.

### 2.11 Safety

AEs will be recorded at each visit during the trial. A SAE refers to 1 of the following AEs:

(1) death or life-threatening condition
(2) necessity of hospitalization or extension of hospitalization period
(3) persistent or meaningful disability or deterioration of function
(4) congenital malformation or abnormalities
(5) other medically important situations.

In case of any event that is considered to have a significant effect on the safety and health status of participants, investigators and relevant experts will determine whether it is considered to be a SAE, and depending upon the medical decision, appropriate action will be taken accordingly.

Safety variables are AEs, vital signs, and laboratory tests. Safety variables will be measured on all the visits during the trial, and their frequency, onset, severity, and causal relationship with the use of drugs will be checked. For each group, the frequency of causal AEs and non-causal AEs will be recorded. Comparisons between groups will be made for the number of AEs and the proportion of participants who have experienced 1 or more AEs. Descriptive statistics will be presented on between-group comparison of vital signs and laboratory tests at before and after the administration of drug and their changes.

### 2.12 Data management

All data will be recorded electronically in electronic case report form. A basic clinical trial document refers to a document that allows individual or overall evaluation of the performance of the trial and the quality of data obtained from it. The basic clinical trial document includes all source documents, monitoring records, correspondence, and regulations. The investigator will keep the protocol, documents, approvals, and all other data related to the clinical trial in the document storage room of the IRB for 3 years from the date of completion. Documents whose retention period expires will be shredded or incinerated. Investigators will have access to the final data sets. Data sets will be housed at https://www.mytrial.co.kr/.

### 2.13 Monitoring

Monitoring will be performed to protect the rights and welfare of participants, to confirm whether the reported data is accurate, complete, and verifiable against source documents, and to ensure whether the clinical trial is being carried out in accordance with approved protocols and applicable regulatory requirements. Monitoring will be conducted by a designated separate company and its staff through regular visits to the institution or over the telephone. When visiting, the monitor will basically check the original record, medication record, and data storage. The monitor will also examine the procedure of the trial and consult with the investigator if there is a problem. The plan includes initiation site visit, interim monitoring visit, and close-out site visit, which will be conducted by visiting the institution in principle.

## 3 Discussion

The current study is planned to examine the effect and safety of *H placenta* treatment on the cognitive abilities and mood of individuals with MCI. To the best of our knowledge, there have been no clinical trials examining the effect of pharmacopuncture on cognitive decline in a population of patients with MCI.

There are some systematic reviews that have evaluated the effect of acupuncture on cognitive function in patients with MCI or dementia. A meta-analysis reported that acupuncture could be effective for Alzheimer disease. Acupuncture combined with donepezil improved the MMSE score and activities of daily living compared to donepezil alone. AEs were observed in 7 out of 3415 cases, demonstrating the low risk.^[38]^ Acupuncture combined with other therapies also improved MMSE scores of patients with vascular MCI.^[39]^

Even though the effect of respective acupoints is not elucidated in clinical studies, the acupoints selected were those most cited in previous studies regarding cognitive function. GV20 and ST36 were used in experiments to observe cell proliferation and neuroblast differentiation in the hippocampus using acupuncture and electroacupuncture in rats.^[40]^ In the experiment applying electroacupuncture in GV20, SAMP8 mice showed improved behavior in the Morris water maze test and changes in metabolite analysis observed using proton nuclear magnetic resonance spectroscopy.^[41]^ Acupuncture treatment in GV20 and 3 other acupoints improved the the Alzheimer's Disease Assessment Scale-Cognitive Subscale (ADAS-cog) score of Alzheimer's disease patients and significantly changed oxidative stress marker levels in cerebrospinal fluid, blood, and urine.^[42]^ Acupuncture treatment was performed on SAMP8 mice in acupoints such as CV12 and ST36, resulting in significant differences in Morris water maze test results, indicating improvement in cognitive function, and changes in cell number, cell proliferation, and the expression of aging-related genes in hippocampal areas such as the dentate gyrus and CA3.^[43–45]^ Another study evaluated the effect of *H placenta* pharmacopuncture treatment in CV12 in Aβ-infused mice and showed behavioral change in the Morris water maze test. It also demonstrated that the expression of intracellular IL-1β and TNF-α was reduced in the hippocampus, and the oxidative stress, microglia, reactive astrocyte, tau and presenilin 1/2 was suppressed.^[19]^ Comparing administering acupuncture in ST36 versus sham acupoints in Alzheimer's disease rat model, blood perfusion and glycol metabolism were found to be increased in the bilateral limbic system, bilateral temporal lobe, right amygdala, and right hippocampus on positron emission tomography scan.^[46]^ Patients with Alzheimer disease were treated using acupoint thread embedding in ST36 and 3 more acupoints once monthly for 6 months (n = 13), and the MMSE and ADAS-cog scores were significantly changed compared to those in the control group (n = 13) (MMSE *P* < .01; ADAS-cog *P* < .05); however, the activities of daily living score did not change (*P* > .05). The functional MRI findings showed that the underlying mechanism might be related to the frontal and temporal lobe, limbic system, as well as cerebellum.^[47,48]^ Electroacupuncture treatment on the same region showed changes in the temporal lobe, limbic system, cerebellum, claustrum, and lobus insularis on functional MRI.^[49]^ In this study, we adopted *H placenta* for pharmacopuncture treatment. Coupled with the effect of *H placenta*, the effect of acupuncture is expected to advance the memory enhancement effect.

We have designed the trial to simultaneously assess mood, sleep quality and quality of life of MCI patients as secondary outcomes. As mood change and sleep disturbance are reported rick factors for progression from MCI to dementia,^[50,51]^ management of mood- and sleep-related symptoms is necessary. In addition to the effect of *H placenta* on cognitive function, its effect on mood and sleep has been previously reported. *H placenta* improved the level of monoamines in brain^[52]^ and reduced depressive-like behavior by modulating oxidative stress.^[53]^ Several case reports regarding the effect of *H placenta* on insomnia have been published.^[54–56]^ Thus, we expect that the auxiliary effect of *H placenta* will be estimated in this trial.

We are suggesting a new administration route of *H placenta* to combine the effect of acupuncture and that of *H placenta* on MCI. By assessing multiple aspects of MCI patients, the data from this proposed study can contribute to expanding the scope of MCI management and prevention of dementia progression.

## 4 Trial status

The study is registered with the Clinical Research Information Service (KCT0005368). This protocol is based on version 1.7 of 05 Aug 2020. First participant will be enrolled in September, 2020. We anticipate that the recruitment will be completed by October, 2020. The data set is summarized in Table 2.

**Table 2:** Items from the World Health Organization trial registration data set.

Data category	Information
Primary Registry and Trial Identifying Number	CRIS, KCT0005368
Date of Registration in Primary Registry	02 Sep 2020
Source(s) of Monetary or Material Support	Korea Health Technology R&D Project through the Korea Health Industry Development Institute (KHIDI), funded by the Ministry of Health & Welfare, Republic of Korea
Contact for Public/ scientific Queries	SC (chosh@khmc.or.kr)
Public Title	None
Scientific Title	Efficacy and safety of Hominis placenta pharmacopuncture on mild cognitive impairment: Randomized, Double blind, Placebo-controlled, Multi-center Trial
Countries of Recruitment	Republic of Korea
Health Condition(s) or Problem(s) Studied	Mild cognitive impairment
Intervention(s)	Active comparator: *Hominis placenta* pharmacopunctureSham comparator: Saline pharmacopuncture
Key Inclusion and Exclusion Criteria	Inclusion criteria: Male and female adults aged 50 to 80, who are diagnosed with mild neurocognitive impairment based on DSM-5, scored CDR 0.5 and voluntarily participated in the trial.Exclusion criteria: diagnosis of dementia, neurological disease, psychiatric diseases.
Study Type	Type of study: InterventionalStudy design: randomized; subject-assessor blind; parallel group; treatmentSealed envelopes; computer-generated random numbers
Date of First Enrollment	05 Sep 2020
Sample Size	30
Recruitment Status	Not yet recruiting
Primary Outcome(s)	MoCA-K
Key Secondary Outcomes	MMSE-DS, K-DRS, CDR, GDS, K-BDI-II, STAI, STAXI, ISI, EQ-5D, EQ-VAS, GQOL-D

## Author contributions

SC is the chief investigator who conceived the study and led the proposal and protocol development. SC, YK, JL, and IJ contributed to the study proposal and overall design. YK and YE developed the protocol. All authors read and approved the final manuscript.

**Conceptualization:** Yunna Kim, Jae Hyok Lee, In Chul Jung, Seung-Hun Cho.

**Investigation:** Yunna Kim, Seung-Hun Cho.

**Methodology:** Yunna Kim, Jae Hyok Lee, Yoon Ji Eom, Seung-Hun Cho.

**Project administration:** Jae Hyok Lee, In Chul Jung, Yoon Ji Eom, Seung-Hun Cho.

**Supervision:** In Chul Jung, Seung-Hun Cho.

**Visualization:** Yunna Kim.

**Writing – original draft:** Yunna Kim, Seung-Hun Cho.

**Writing – review and editing:** Yunna Kim, Jae Hyok Lee, In Chul Jung, Yoon Ji Eom, Seung-Hun Cho.

## Supplementary Material

Supplemental Digital Content

## Supplementary Material

Supplemental Digital Content
