# Supplementary material for: Efficacy and safety of Hominis placenta pharmacopuncture on mild cognitive impairment: Randomized, double blind, placebo-controlled, multi-center trial
Source: Medicine (Baltimore). 2020 Nov 13;99(46):e22956. doi: 10.1097/MD.0000000000022956 (PMC7668472; doi:10.1097/MD.0000000000022956)
Supplement: Supplemental Digital Content [file medi-99-e22956-s002.docx]

Additional file 2. Organizational structure and responsibilities

| Principal Investigator and  Research Physician | Trial Management Committee (TMC)  (Principal investigator, Research Physician, Administrator) | Data Manager |
| --- | --- | --- |
| Design and conduct of the trial  Preparation of protocol and revisions  Preparation of CRFs (Case Report Forms)  Organizing trial management committee meetings  Managing CTO [Clinical Trials Office]  Publication of study reports  Members of TMC [Trial Management Committee]  Agreement of final protocol | Study planning  Reviewing progress of study and if necessary agreeing changes to the protocol  Provide annual risk report to ethics committee  SUSAR (Serious unexpected suspected adverse events) reporting  Responsible for trial master file  Budget administration and contractual issues  Data verification  Randomization  Data collection | Maintenance of trial IT system and data entry  Data verification |
